# [Corrigendum] Expression of FKBP52 in the ovaries of PCOS rats

**DOI:** 10.3892/ijmm.2026.5850

**Published:** 2026-05-11

**Authors:** Shiyan Song, Yong Tan

Int J Mol Med 43: 868-878, 2019; DOI: 10.3892/ijmm.2018.3998

Following the publication of the above article, an interested reader drew to the authors' attention that, concerning the images showing the green fluorescence of granulosa cells in Fig. 5 on p. 874, the images selected for Fig. 5B and C were apparently matching, albeit with different levels of brightness, even though the experimental conditions for these figure parts were reported to be different.

Upon investigating this figure, the authors realized that the image correctly shown for Fig. 5C had also inadvertently been included as Fig. 5B. A revised version of Fig. 5, now showing the correct data panel for Fig. 5B, is shown below. The authors confirm that the error made during the assembly of this figure did not have any significant impact on either the results or the conclusions reported in this study, and all the authors agree with the publication of this Corrigendum. The authors are grateful to the Editor of *International Journal of Molecular Medicine* for allowing them the opportunity to publish this Corrigendum; furthermore, they apologize to the readership of the Journal for any inconvenience caused.

## Figures and Tables

**Figure 5:**
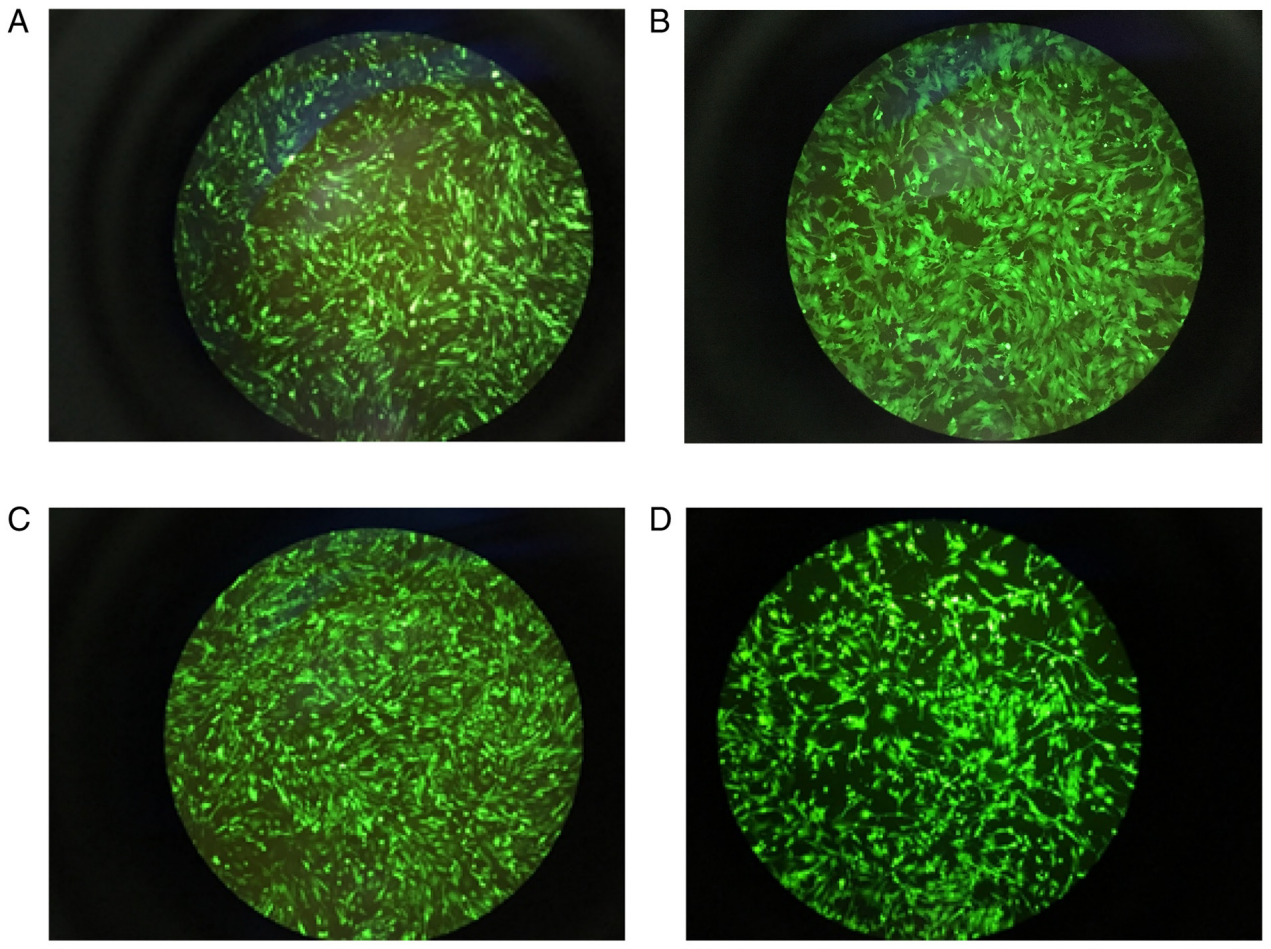
Expression of green fluorescence in GCs. (A) Oe negative control virus group, 10^11^ PFU/ml, MOI=200; (B) FKBP4-Oe virus group, 2×10^10^ PFU/ml, MOI=400; (C) RNAi negative control virus group, 5×10^10^ PFU/ml, MOI=100; (D) FKBP4-RNAi virus group, 2×10^10^ PFU/ml, MOI=400). The cell transfection rate was >80%. Magnification, ×100. GCs, granulosa cells; MOI, multiplicity of infection.

